# Adjuvanted influenza vaccine dynamics

**DOI:** 10.1038/s41598-018-36426-9

**Published:** 2019-01-11

**Authors:** César Parra-Rojas, Veronika von Messling, Esteban A. Hernandez-Vargas

**Affiliations:** 1 Frankfurt Institute for Advanced Studies, 60438 Frankfurt am Main, Germany; 2 Veterinary Medicine Division, Paul-Ehrlich-Institut, Federal Institute for Vaccines and Biomedicines, 63225 Langen, Germany

## Abstract

Adjuvanted influenza vaccines constitute a key element towards inducing neutralizing antibody responses in populations with reduced responsiveness, such as infants and elderly subjects, as well as in devising antigen-sparing strategies. In particular, squalene-containing adjuvants have been observed to induce enhanced antibody responses, as well as having an influence on cross-reactive immunity. To explore the effects of adjuvanted vaccine formulations on antibody response and their relation to protein-specific immunity, we propose different mathematical models of antibody production dynamics in response to influenza vaccination. Data from ferrets immunized with commercial H1N1pdm09 vaccine antigen alone or formulated with different adjuvants was instrumental to adjust model parameters. While the affinity maturation process complexity is abridged, the proposed model is able to recapitulate the essential features of the observed dynamics. Our numerical results suggest that there exists a qualitative shift in protein-specific antibody response, with enhanced production of antibodies targeting the NA protein in adjuvanted versus non-adjuvanted formulations, in conjunction with a protein-independent boost that is over one order of magnitude larger for squalene-containing adjuvants. Furthermore, simulations predict that vaccines formulated with squalene-containing adjuvants are able to induce sustained antibody titers in a robust way, with little impact of the time interval between immunizations.

## Introduction

Seasonal and pandemic influenza A virus (IAV) infections pose a serious threat to public health. Influenza readily spreads across borders, and can affect several countries simultaneously, resulting in considerable economic and social impact. Seasonal outbreaks cause millions of infected cases and about half a million deaths worldwide every year^1,2^. Furthermore, the consequences of epidemics can be economically devastating, since they can also affect susceptible poultry and swine populations.

Vaccines represent a cornerstone of measures against influenza outbreaks; however, a variety of important limitations exist in terms of the availability, cost and effectiveness of currently licensed influenza vaccines. A comprehensive quantitative evaluation of the within-host effects of vaccination is still lacking, and the elaboration of vaccination strategies that overcome these difficulties remains a fundamental challenge^3^.

Influenza A viruses are classified into subtypes according to the antigenicity of their two main surface glycoproteins: hemagglutinin (HA) and neuraminidase (NA). The former is responsible for virus entry by binding to sialic acids on the surface of hosts cells and subsequent pH-dependent fusion of the viral and endosomal membranes, while the latter mediates the release of newly produced virions from infected cells by removing sialic acid from their surfaces^4–6^. Due to these different functions, neutralizing antibodies are primarily directed against the HA protein^7^. The antibody response directed against NA, in turn, plays a role in decreasing viral spread by provoking the accumulation of virus on the cell surface, which reduces morbidity and mortality in mice^8,9^.

Antibody responses against the virus drive antigenic drift, which consists in gradual changes to the surface proteins HA and NA. Occasionally, reassortment may lead to the introduction of a new HA or NA segment—also referred to as antigenic shift—resulting in the apparition of entirely novel strains, for which the population is immunologically naive, with potentially severe global consequences^6,10^. To date, 18 HA and 11 NA subtypes have been identified, with only a few of them—H1, H2, and H3 and N1 and N2, respectively—found in human seasonal viruses^11^. Within a given subtype, the mutation rate in NA is lower than that in HA^12^—that is, NA is more *antigenetically conserved*—possibly owing to the fact that the antibody response is skewed towards HA, resulting in a greater selection pressure^6,13^.

As a consequence of antigenic drift, the strain composition in seasonal vaccines has to be updated regularly^14^. This is a costly endeavor that, at the same time, does not address the latent threat of further antigenic drift or a pandemic caused by a newly reassorted strain, since the vaccines are highly strain-specific. There is thus a need for immunization strategies that can elicit a broad immune response; specifically, the production of broadly cross-reactive antibodies that confer protection from strains of the virus different from those present in the vaccine.

In pandemic situations, when a large number of doses is needed in a very short time^15^, limited antigen availability is an additional major challenge. This may be addressed by antigen-sparing strategies in combination with adjuvants, which trigger a strong immune response at lower antigen doses than otherwise necessary^16^. Interestingly, the addition of squalene-containing adjuvants has also been observed positively influence the breadth of the antibody response by enhancing not only the overall titers but also the production of antibodies that target the NA protein^17^. Since NA is more conserved, antibodies directed towards this protein may confer partial immunity against other influenza strains carrying the same NA subtype^18^.

Mathematical models of biological processes can yield insight on their most essential features, as well as contribute in delineating experimental studies. While a variety of models exists that explore the within-host dynamics of influenza infection, most mathematical descriptions of influenza are constructed at the population level^19^. In particular, those incorporating vaccination are mainly concerned with the epidemiological consequences of different vaccination strategies in a given population—see, *e*.*g*., Weycker *et al*.^20^, and van den Dool *et al*.^21^.

Inside the host itself, antibody production is driven by a process referred to as *affinity maturation* (AM), whereby B cells undergo several rounds of proliferation, mutation and selection within specialized domains called germinal centers (GCs) towards increasing binding affinities to the antigen, ultimately differentiating into high-affinity antibody-secreting plasma cells and memory B cells^22^. The dynamics inside the GCs are essential to the strength and cross-reactivity of antibody responses to infection or vaccination, and have received considerable attention, both conceptually^22–24^ and from a modeling perspective to various degrees of detail^25,26^. These models have also incorporated the effects of different vaccination strategies on the AM process, notably for the cases of malaria^27^ and HIV^28,29^.

In this work, we construct a mathematical model to capture the within-host effects of immunization with adjuvanted and non-adjuvanted influenza vaccine formulations. By means of a parsimonious description of the AM process, we predict the magnitude and protein-specificity of the antibody response elicited by the different vaccine formulations at a coarse level. Using the data from Schmidt *et al*.^17^, we fit our model parameters to virus-neutralizing antibody titers, and qualitatively compare our results to the experimentally observed protein-specific titers. Our simulation results show that this simple model is able to recapitulate the essential features of antibody production elicited by adjuvanted vaccine formulations, and may serve as a stepping stone for more complex analyses.

## Methods

### Experimental data

For the proposed mathematical modeling analysis, the experimental data previously published by Schmidt *et al*.^17^ is employed. Briefly, adult male and female ferrets, immunologically naive to circulating influenza strains, were immunized intramuscularly with adjuvanted and non-adjuvanted formulations of the inactivated pandemic H1N1pdm09 vaccine, followed by a boost 3 weeks later. The adjuvants tested were the squalene-containing MF59 and AS03, as well as Diluvac Forte, which contains only vitamin E.

Post vaccination, antibody titers against total influenza virus, as well as specific to HA or NA, were determined for a series of different HA and NA proteins. Thus, both the magnitude and specificity of the immune response for the different vaccine formulations were measured. In our specific case, the output of the model outlined in the following section was fitted to the data for functional virus-neutralizing antibodies.

### Mathematical model of antibody production

**Figure 1:**
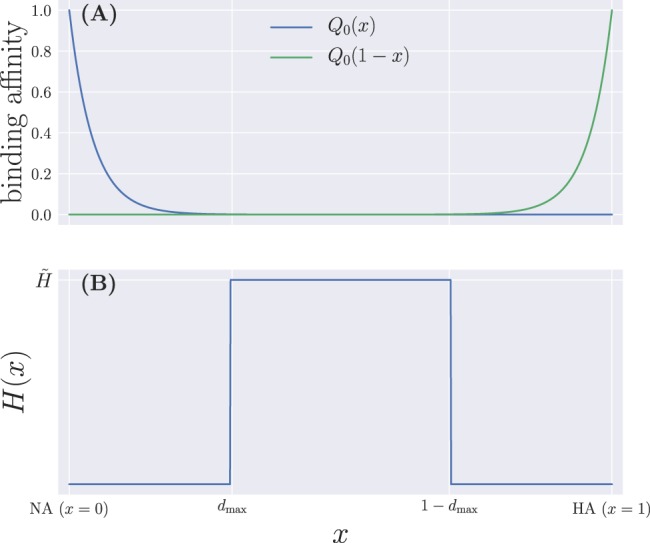
Schematic illustration of the one-dimensional system. B cells and Abs are positioned on the *x*-axis, with NA and HA situated at the extremes of the shape-space. Mutations occur as a diffusion along *x* and are symmetric, there being no selective pressure towards either of the two proteins. (**A**) NA and HA with their affinity curves, respectively given by the functions *Q*_0_(|*x* − 0|) = *Q*_0_(*x*) (blue line) and *Q*_0_(|*x* − 1|) = *Q*_0_(1 − *x*) (green line), determining how affine a cell is to a given protein based on its proximity to either extreme of the interval. The sum of these two quantities wields the total unweighted affinity $\tilde{Q}(x)$ for a given position in shape-space. (**B**) The ‘pool’ of naive B cells, *H*(*x*), extending over all the zero-affinity region and the entrance of the low-affinity region, delimited by the cutoff *d*_max_.

We propose a two-epitope model in a Euclidean, one-dimensional shape space, with the genotype of a given cell represented by its position $x\in [0,1]$. The two epitopes correspond to the NA and HA proteins of the vaccine strain, and will be located at different, non-overlapping positions—*i*.*e*., their antigenic distance is larger than some specified cutoff—in shape space. Without loss of generality, we take NA to be located at position *x* = 0, while HA sits at *x* = 1—see Fig. 1A.

The mathematical model draws mainly from the detailed AM simulations of Chaudhury *et al*.^27^. Our interest, however, lies in the broader effects of the inclusion of adjuvants in the influenza vaccine, and not in the fine details of the AM process. For this reason, the variables we consider explicitly are only two, and correspond to the distributions of B cells and antibodies in the shape space, which we represent by *B*(*x*, *t*) and *Ab*(*x*, *t*), respectively. In other words, we do not distinguish between stimulated B cells, non-stimulated B cells, plasma and memory cells, but rather consider an effective behavior inside a GC, represented by the variable *B*(*x*, *t*). The choice of a simplified shape space, in turn, is inspired by the notion of ‘antibody landscape’ introduced by Fonville *et al*.^30^. In this case, in contrast to the original formulation, it is considered that the points along the axis do not correspond to different virus strains, but rather to the individual virus glycoproteins, as described above.

AM is an inherently stochastic process, and it has been shown that stochasticity plays a decisive role in the selection of clonal lineages in the GC^26^. However, we stress that our interest is not in tracking individual B cells nor in the actual number of B cell genotypes in the system, but rather the broad shape—with respect to protein specificity—and size of the resulting antibody distribution in shape space, and its comparison to the experimental data for Ab titers. Therefore, we consider the case in which there exists a one-to-one correspondence between cell genotype and affinity, as shown below. Due to this, random drift does not play a role in our system—cells with the same affinity as treated as belonging to the same lineage—and the stochastic effects in our model arise predominantly from B cell mutations, which are small in magnitude. We thus turn to a deterministic description of the process, in which the positions of cells in shape space, and their corresponding affinities, are continuous variables, and we choose to represent mutations by an effective diffusion along the *x*-axis.

**Figure 2:**
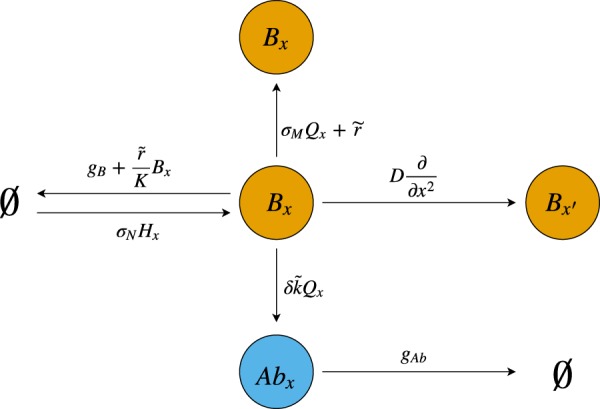
Diagram illustrating the dynamics of Eqs (1) and (2). Here, the symbol for the empty set, $\varnothing$, represents everything that is outside of the system we are considering. There exists a constant influx of B cells at position *x* from a pool of naive cells given by *H*(*x*)—see Eq. (8)—occurring at rate *σ*_*N*_. Base proliferation occurs at a rate $\tilde{r}$, while an additional term representing birth-limited selection is given by a proliferation weighted by the affinity *Q*(*x*) at rate *σ*_*M*_. Cells at position *x* can further produce offspring at a different position *x*′ due to mutations represented by a diffusion coefficient *D*. Natural death of B cells occurs at a rate *g*_*B*_, and a further decay is the result competition for limited resources in the GC, whose carrying capacity is given by *K*. Proliferation and diffusion terms are further modulated by the function *G*(*t*), representing GC activity and given by Eq. (7). A fraction *δ* of B cells at position *x* produce antibodies at position *x* at rate $\tilde{k}$, weighted by their affinity *Q*(*x*), which subsequently decay at a rate *g*_*Ab*_.

We examine two different forms for the time evolution of the distribution of B cells in shape space which we denote, respectively, by model A and model B. The former is given by

1 $$\begin{array}{rcl}\tfrac{\partial B(x,t)}{\partial t} & = & G(t)[{\sigma }_{N}H(x)+{\sigma }_{M}Q(x)B(x,t)+\tilde{r}B(x,t)+D\tfrac{{\partial }^{2}B(x,t)}{\partial {x}^{2}}]\\  &  & -\,{g}_{B}(x)B(x,t)-\frac{\tilde{r}}{K}{[B(x,t)]}^{2},\end{array}$$

2 $$\frac{\partial Ab(x,t)}{\partial t}=\delta \,\tilde{k}\,Q(x)B(x,t)-{g}_{Ab}Ab(x,t),$$

while the explicit form of model B is shown in Eqs (S5) and (S6) of the Supplementary Material. The dynamics above are illustrated in Fig. 2.

Let us start by addressing the growth terms in Eq. (1), and leaving the function *G*(*t*) aside for the time being. The ‘birth’ of B cells comprises three different types of events. First, a constant influx, at a rate *σ*_*N*_, from a pool of low-affinity naive cells, which we represent by a function *H*(*x*) that is only non-zero in low- and zero- affinity regions of the interval $[0,1]$, as shown in Fig. 1B. Binding affinity between a B cell and a protein at antigenic distance *x* is represented by a function *Q*_0_(*x*) that takes values between 0 and 1. Following the model proposed by Chaudhury *et al*.^27^, we take:

3 $${Q}_{0}(x)=\{\begin{array}{r}1\\ {\varepsilon }^{{d}_{{\rm{\min }}}-x}\\ 0\end{array}\,\begin{array}{l}x < {d}_{{\rm{\min }}}\\ {d}_{{\rm{\min }}}\le x\le {d}_{{\rm{\max }}}\\ x > {d}_{{\rm{\max }}}\end{array},$$

where *d*_min_ and *d*_max_ represent cutoffs for maximum and zero affinity, respectively. This means that *d*_max_ will determine the range of influence of *H*(*x*).

Apart from the influx of naive B cells, we represent the recruitment of ‘memory’ cells—unaccounted for in the model—as well as selection by allowing high-affinity B cells to further proliferate at a rate *σ*_*M*_ over the base rate; in the equation, *Q*(*x*) represents the total binding affinity of cells at position *x*, weighted by antigen immunogenicity. In other words,

4 $$Q(x)={\gamma }_{{\rm{NA}}}{Q}_{0}({x}_{{\rm{NA}}})+{\gamma }_{{\rm{HA}}}{Q}_{0}({x}_{{\rm{HA}}}),$$

where *γ*_NA_ and *γ*_HA_ are the immunogenicities of NA and HA, respectively, while *x*_NA_ and *x*_HA_ correspond to the antigenic distances to NA, ${x}_{{\rm{NA}}}\equiv |x-0|=x$, and to HA, ${x}_{{\rm{HA}}}\equiv |x-1|=1-x$, respectively. Finally, affinity-independent B cell proliferation happens at a rate $\tilde{r}$, and the position of the daughter cell in shape space will be given by that of the parent cell plus a normally-distributed random number with variance *ν*. In the mean-field limit, this translates into an overall diffusion coefficient $D=\tilde{r}\nu /2$^31^.

The decay of B cells occurs at a rate *g*_*B*_(*x*); the specific form of this function is chosen so that high-affinity B cells possess a longer lifespan, in order to account for memory cells. We formulate this term as follows:

5 $${g}_{B}(x)=\frac{{g}_{B}^{(0)}}{1+{g}_{B}^{(1)}\tilde{Q}(x)},$$

where $\tilde{Q}(x)$ is the *unweighted* total binding affinity of cells at position *x*, *i*.*e*., $\tilde{Q}(x)\equiv {Q}_{0}(x)+{Q}_{0}(1-x)$. The lifespan of memory B cells is a subject of debate, and there exists evidence for it ranging from months to years^32,33^. Here, we set ${g}_{B}^{\mathrm{(1)}}$ so that B cells have a maximum possible mean life of 2 years. We note that what we have taken to represent the presence of memory B cells in the system in the equation above may be also regarded as a form of *death-limited selection*; conversely, the selection term in Eq. (1) corresponds to *birth-limited selection*. These concepts have been recently introduced by Chakraborty *et al*.^26^, who explored the influence of both types of selection in the loss of clonal diversity in the GC during the AM process.

Finally, the GC has a finite carrying capacity *K*, and B cells with similar genotypes will compete for space in the system^23^. The competition will be in general non-local and can be written in the form

6 $$\frac{\tilde{r}}{K}B(x,t)\,{\int }_{0}^{1}\,{\rm{d}}y\,{\mathscr{C}}(|x-y|)B(y,t).$$

The exact form of the competition kernel, ${\mathscr{C}}(x)$, will determine the strength and range of this competition. For the sake of simplicity, we consider a Dirac-*δ* kernel; that is, the competition is made completely local and assumed to have unit strength. This yields the last term in Eq. (1).

The difference between models A and B is given by the modulation function *G*(*t*). This corresponds to a general measure of activity of the immune response, which we assume to vary between 0 and 1 for inactive and fully active, respectively; that is, *G* can be regarded as a function that determines whether affinity maturation is taking place. We explicitly choose not to identify this function with the concentration of free antigen in the system, due to the fact that the formation of the GC is dependent on antigen presence, but not on antigen quantity; once active, its subsequent dynamics and decay are essentially antigen-independent^24^.

In the case of model A, the activity level governs all processes resulting in the growth of the B cell population, whereas model B assumes that only recruiting events—of naive and of ‘memory’ B cells—are affected by it, permitting the free proliferation of B cells as long as a non-zero quantity remains in the system. In other words, in Eq. (1) *G* encompasses the activity of the response as a whole, from antigen uptake and B cell migration into the GC to proliferation inside the GC itself. In Eq. (S5), in contrast, only antigen uptake and cell recruitment are affected by *G*. The activity level is assumed to decay exponentially with a rate *μ*, so that *G* takes the form

7 $$G(t)={e}^{-\mu (t-{t}_{k})},\,{t}_{k}\le t < {t}_{k+1},$$

where *t*_0_ = 0 corresponds to moment of initial immunization and the *t*_*k*_, *k* = 1, 2, …, to the subsequent boosts. In this case, the animal experiments were performed with a single immunization boost at *t*_1_ = *t*_boost_ = 21 days. Again, since *G*(*t*) does not represent the antigen concentration, its value is not affected by the presence of antibodies in the system.

The equation describing antibody dynamics, Eq. (2), is simpler, consists of an antibody production term, mediated by weighted B cell affinity to the antigen, and a constant exponential decay rate. Since not all B cells end up producing antibodies, but only the ones among them that differentiate into plasma cells, we multiply the production term by an attenuating factor *δ*. We choose to take this factor as the differentiation probability from Chaudhury *et al*.^27^.

Given a non-negative initial condition, *B*(*x*, 0) ≥ 0 and *Ab*(*x*, 0) ≥ 0, the solutions to Eqs (1) and (2) will remain non-negative for all *t* > 0. This can be seen from the fact that both *G*(*t*) and *H*(*x*) are non-negative for all *t* and all *x*, respectively, so that ∂_*t*_*B* ≥ 0 for *B* → 0; as a consequence of this, we obtain ∂_*t*_*Ab* ≥ 0 for *Ab* → 0, since *Q* is also non-negative for all *x*. Furthermore, the non-linear term in Eq. (1), stemming from the finite carrying capacity *K*, ensures that the solution for *B* remains bounded for all *x*, *t*, which, in turn, yields ∂_*t*_*Ab* < 0 for large values of *Ab*. A more formal mathematical analysis of the character of the solutions to Eqs (1) and (2) is beyond the scope of this paper.

We note that, in absence of the decay in GC activity signified by *G*(*t*), these equations admit non-zero steady-state solutions. The solution for *B* is characterized by a constant non-zero value in the zero-affinity region, with peaks corresponding to B cells that have some degree of specificity towards either of the glycoproteins. Since only non-zero affinity B cells end up producing antibodies, the solution for *Ab* will be non-zero only towards the edges of the interval, vanishing everywhere else. In both cases, the profiles of the left- and right-most portions of the distribution will be modulated by the affinity *Q*(*x*), with higher-affinity cells being more abundant in the steady state. In our particular case, however, the modulation introduced by *G*(*t*) has the effect of ultimately yielding solutions for both *B* and *Ab* that vanish in the entire interval as *t* → ∞.

#### Parameters

Despite the fact that we have attempted to keep the model simple, it still contains a large number of parameters. Due to this, we have decided to fix most of them and focus on those that govern the differences between vaccine formulations. The fixed parameters values in the model are primarily taken from Chaudhury *et al*.^27^, and we have kept their labels whenever appropriate. In the cases when a single event in our model encompasses multiple events in the original one, we have assumed that the time it takes for this event to happen is an exponentially distributed variable with a mean equal to the sum of the mean waiting times of the original events; that is, the mean time for a B cell to proliferate is given by the mean stimulation time 1/*σ*_*B*_ plus the mean proliferation time of stimulated B cells 1/*r*, thus the resulting proliferation rate $\tilde{r}={\sigma }_{B}r/({\sigma }_{B}+r)$. In the same manner, the effective antibody production rate $\tilde{k}$ is obtained from the B cell stimulation rate *σ*_*B*_ and the antibody production rate of plasma cells *k*_*Ab*_ as $\tilde{k}={\sigma }_{B}{k}_{Ab}/({\sigma }_{B}+{k}_{Ab})$—recall that the probability of differentiation into plasma cells is explicitly included in Eq. (2).

Furthermore, we choose to fix *d*_min_ = 0 for simplicity, which amounts to neglecting degeneracy in the number of B cell genotypes presenting full binding affinity to the glycoproteins^27^—we expect that a *d*_min_ > 0 would have a negligible effect in the model output—while we set *ε* = 10^10^. This latter choice is based on the fact that for a Euclidean shape space to match immunological data, the normalized stimulation radius can be chosen to lie somewhere between 0.15 and 0.22^34^, and we adjust the value of *ε* in order to allow a 10–10^3^-fold increase in binding affinities from naive to fully mature B cells^27^, with the exact number determined by the value of *d*_max_. We also fix a small value for the mutational variance—in this case, *ν* = 10^−4^, so that a mutation of the order of one standard deviation translates into a shift of the order of one percent in genotype space—and the width of *H*(*x*) so that the pool of naive B cells occupies one percent of the non-zero affinity zone on each side of the shape space. More explicitly, we have

8 $$H(x)=\{\begin{array}{l}\tilde{H}\\ 0\end{array}\,\begin{array}{c}0.99\,{d}_{{\rm{\max }}}\le x\le 1-0.99\,{d}_{{\rm{\max }}}\\ {\rm{otherwise}}\end{array},$$

where $\tilde{H}$ is the height of the pool, which we have set to $\tilde{H}=100$ cells. These choices are further commented on in the Discussion section.

All fixed parameters in the model are listed in Table 1. The protein immunogenicities, the affinity cutoff and the GC activity decay rate are free parameters.

**Table 1:** Fixed parameters appearing in the model defined by Eqs (1) and (2).

Parameter	Value	Notes
*σ*_*N*_	1.0 [days^−1^]	from ref.^27^
*σ*_*M*_	1.0 [days^−1^]	from ref.^27^
$\tilde{r}$	1.5 [days^−1^]	derived from *σ*_*B*_ and *r* in ref.^27^
*K*	5000 [cells]	from ref.^27^
${g}_{B}^{(0)}$	0.22 [days^−1^]	from ref.^27^
$\tilde{k}$	0.75 [days^−1^]	derived from *σ*_*B*_ and ub in ref.^27^
*g*_*Ab*_	0.1 [days^−1^]	from ref.^27^
*δ*	0.1	from ref.^27^
*t*_boost_	21 [days]	interval between immunizations in the experiments
${g}_{B}^{(1)}$	161.22	based on refs^32,33^
*ε*	10^10^	based on refs^27,34^
*d*_min_	0.0	fixed for simplicity
*ν*	10^−4^	rescales with $\tilde{H}$, fixed for simplicity
$\tilde{H}$	100 [cells]	rescales with *ν*, fixed for simplicity

Derived values correspond to considering a series of events as a single joint event—B cell stimulation and proliferation taken together as proliferation, for $\tilde{r}$, and B cell stimulation and Ab production taken together as Ab production, for $\tilde{k}$. We fix ${g}_{B}^{(1)}$ to allow for a maximum mean life of 2 years for long-lived B cells^32,33^, while the value of *ε* stems from shape-space considerations^27,34^. Other parameters are fixed to simplify the model; however, we do not expect them to have a large impact on the conclusions drawn from it, since all of the parameters listed here remain constant across vaccine formulations, and our focus are the differences between them and the mechanisms driving them—see the Parameters section and the Discussion. In this case, *d*_max_, *γ*_NA_, *γ*_HA_, *μ*, *β*_NA_, *β*_HA_ and *β*_*Ab*_ are free parameters to be determined.

#### Effects of adjuvants

The main features of the experimental data correspond to enhanced antibody response and enhanced response towards the NA protein for the adjuvanted vaccine formulations, as opposed to the non-adjuvanted case. We introduce these effects in the form of factors *β*_*i*_—which are free parameters in the model—modifying the immunogenicities and antibody production parameters as follows:

9 $${\gamma }_{{\rm{NA}}}\to {\beta }_{{\rm{NA}}}\,{\gamma }_{{\rm{NA}}},$$

10 $${\gamma }_{{\rm{HA}}}\to {\beta }_{{\rm{HA}}}\,{\gamma }_{{\rm{HA}}},$$

11 $$\tilde{k}\to {\beta }_{Ab}\,\tilde{k}.$$

Here, all *β*_*i*_ ≥ 1, with the equality holding in the non-adjuvanted case. The different values *β*_*i*_ regulate the strength of the effects and are adjuvant-dependent. We expect that *β*_NA_ > *β*_HA_, reflecting a selection pressure towards the production of NA-specific antibodies.

### Parameter estimation

The free parameters in the model, as described above, correspond to the affinity cutoff *d*_max_, the base immunogenicities *γ*_NA_ and *γ*_HA_, the decay rate in GC activity *μ*, and the adjuvanticity factors *β*_NA_, *β*_HA_ and *β*_*Ab*_. The first four parameters in this list can be estimated from the data for the non-adjuvanted formulation of the vaccine, while the other three can be obtained for each adjuvant in the data set after fixing these.

Specifically, we fit the output of the model defined by Eqs (1) and (2), *y*^(model)^, to the data from Schmidt *et al*.^17^ corresponding to the virus-neutralizing antibody titers as a function of time following vaccination and immunization boost. This is compared to the total area under the curve of the distribution given by *Ab*(*x*, *t*); that is, ${y}^{({\rm{model}})}(t)=\int \,{\rm{d}}x\,Ab(x,t)$. We use the Differential Evolution (DE) algorithm^35^ to estimate the best parameter values by minimizing the root mean squared error (RMSE). The cost function takes the form

12 $$RMS{E}_{{\rm{\Theta }}}={[\frac{1}{N}\sum _{i=1}^{N}{({y}_{{\rm{\Theta }}}^{({\rm{model}})}(t={t}_{i})-{y}_{i}^{({\rm{data}})})}^{2}]}^{1/2},$$

where *i* runs over all the *N* individual points in the data set, and Θ corresponds to a particular set of parameters. In other words, we aim to find the optimal set Θ* that minimizes *RMSE*_Θ_. As mentioned above, this process is carried out in two steps, with

13 $${{\rm{\Theta }}}^{{\rm{base}}}=\{{d}_{{\rm{\max }}},{\gamma }_{{\rm{NA}}},{\gamma }_{{\rm{HA}}},\mu \},$$

14 $${{\rm{\Theta }}}^{{\rm{adjuvants}}}=\{{\beta }_{{\rm{NA}}},{\beta }_{{\rm{HA}}},{\beta }_{Ab}\}.$$

We note that we could have, alternatively, minimized the mean squared error (MSE) or residual sum of squares (RSS) yielding equivalent results, the advantage of the RMSE being that it has interpretable units: in this case, the error is given in units of Ab titer.

The equations themselves are solved using no-flux boundary conditions, and with initial condition *B*(*x*, 0) = 0 and *Ab*(*x*, 0) = 1; the latter due to the fact that the first data point for all vaccine formulations satisfies ${y}_{0}^{({\rm{data}})}=1$. We use a grid of *N*_*x*_ = 1500 points, with locations given by ${\rm{d}}x/2,3\,{\rm{d}}x/2,\ldots ,1-{\rm{d}}x/2$, where ${\rm{d}}x\equiv 1/{N}_{x}$.

The best fit parameters, along with the bounds used for them in the estimation procedure, are shown in Table 2 for model A, and Table S2 for the case of model B. We have given *γ*_HA_ more freedom of movement than *γ*_NA_, due to the fact that HA is more immunogenic than NA^13^; however, we have not made *γ*_HA_ > *γ*_NA_ a hard constraint. Instead, in order to reflect the imbalance between immunogenicities we have made the upper bound for *γ*_HA_ in the estimation three times larger than that of *γ*_NA_—see Table 2.

**Table 2:** Best fit parameters for model A.

Parameter	bounds	best overall fit	mean	median	std	min	max
**Non**-**adjuvanted**							
*d*_max_	(0.1, 0.3)	0.1058	0.1839	0.1610	0.0716	0.1	0.2784
*μ*	(0.2, 1.0)	0.4602	0.2970	0.2285	0.1312	0.2	0.7155
*γ*_NA_	(0.1, 2.5)	2.4345	1.8367	2.3615	0.8673	0.1	2.5
*γ*_HA_	(0.1, 7.5)	7.0999	6.7012	7.4251	1.5994	0.1509	7.5
**MF59**							
*β*_NA_	(1.0, 5.0)	1.7124	1.6327	1.6016	0.3376	1.0042	4.9972
*β*_HA_	(1.0, 5.0)	1.0039	1.0217	1.0015	0.1391	1.0	3.5700
*β*_*Ab*_	(1.0, 60.0)	17.6365	17.2731	13.2060	10.3760	1.0008	44.6023
**AS03**							
*β*_NA_	(1.0, 5.0)	1.7716	2.1502	1.6285	1.1580	1.0	5.0
*β*_HA_	(1.0, 5.0)	1.0239	1.2691	1.0060	0.5701	1.0	4.0200
*β*_*Ab*_	(1.0, 60.0)	52.2757	23.8041	18.0365	17.2371	1.0	60.0
**Diluvac**							
*β*_NA_	(1.0, 5.0)	1.5998	1.2906	1.0720	0.4464	1.0	4.4580
*β*_HA_	(1.0, 5.0)	1.0031	1.1070	1.0002	0.2659	1.0	1.7942
*β*_*Ab*_	(1.0, 60.0)	1.3936	1.2612	1.0021	0.6167	1.0	3.4398

The best overall fit corresponds to estimating the parameters using the full dataset, while the statistical properties are the result of performing the estimation on 2500 bootstrapping samples of the data. The samples are constructed by selecting, at random, one datapoint per timepoint in the original dataset.

Additionally, Table 2 also shows the results of carrying out the parameter estimation on 2500 bootstrapping samples from the data, constructed by selecting, at random, one datapoint per timepoint in the original dataset. As for the case discussed above, the parameters for the adjuvanted vaccine formulations are estimated after fixing the values of parameters from the non-adjuvanted case to those providing the best fit when considering all datapoints, *i*.*e*., the values from Table 2. There exists large variability on the estimates and, in particular, we see that the immunogenicities tend to explore their full range of values, which we understand as a consequence of the fact that these two parameters are generally expected to rescale one another—see the Discussion for more details on this.

Finally, in order to evaluate the models, we employ a baseline consisting in a non-spatial version of model A, *i*.*e*., without taking protein-specificity into account, which we denote by model 0. In this case, we replace all *x*-dependent terms by scalar ones, so that *H*(*x*) becomes *H* and represents a naive influx rate, while *Q*(*x*) becomes an effective measure of affinity *Q*—see Eqs (S1) and (S2) for details. Table 3 compares the models to this baseline in terms of their goodness of fit and complexity, showing both the RMSE and Akaike Information Criterion (AIC)^36^. In general, while qualitative differences between the models are easily observable, as discussed below, a quantitative evaluation based on AIC clearly favors model A only for the first part of the sequential parameter estimation, corresponding to the non-adjuvanted vaccine formulation, for which its performance is equivalent to the baseline. For the adjuvanted cases, models A and B—as well as C, introduced below—behave similarly.

**Table 3:** RMSE and AIC for all models considered.

Model	RMSE	AIC
**Model 0**		
non-adjuvanted	47.2175	223.8668
MF59	1870.1953	425.8927
AS03	5038.3010	481.3902
Diluvac	92.8236	257.7192
**Model A**		
non-adjuvanted	47.0733	223.6956
MF59	2146.2261	435.6021
AS03	5333.6525	486.5803
Diluvac	97.3368	262.3779
**Model B**		
non-adjuvanted	58.0708	235.4531
MF59	1956.1513	430.4091
AS03	5354.4737	486.7985
Diluvac	102.3938	265.2143
**Model C**		
MF59	2227.4190	435.6815
AS03	5672.4353	488.0289
Diluvac	97.8185	260.6544

Details of models 0, B and C can be found in the Supplementary Material.

All code is implemented in Python and is freely available at: https://github.com/systemsmedicine/adjuvanted-vaccine.

## Results

### Kinetics of antibody response

**Figure 3:**
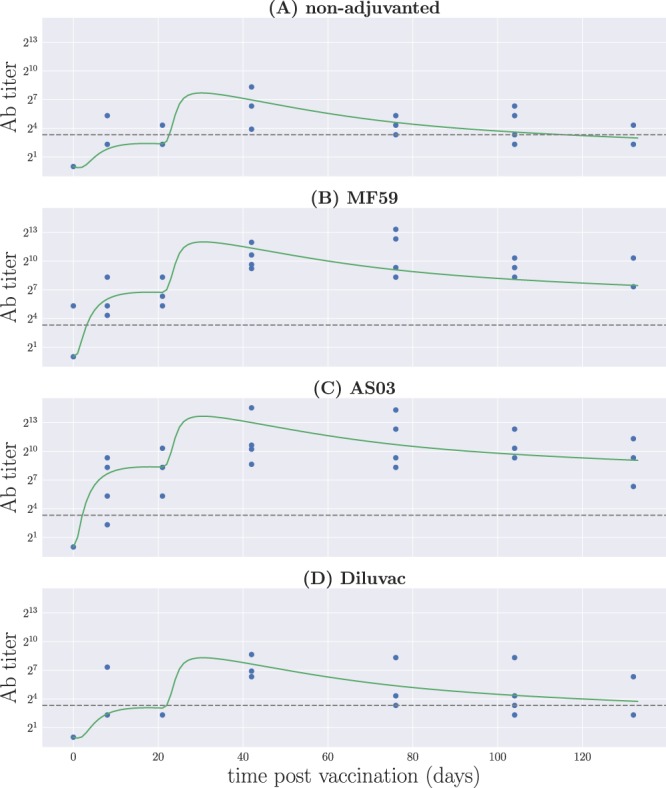
The raw data for virus-neutralizing antibody titers, compared to the area under the curve of *Ab*(*x*, *t*) resulting from model A for the best parameter values, Θ*. (**A**) Non-adjuvanted case; (**B**) MF59; (**C**) AS03; (**D**) Diluvac. In all cases, the gray, dashed line shows the detection limit for neutralizing antibodies.

Figure 3 shows the output from model A as given by the best fit parameters for the non-adjuvanted and all three adjuvanted formulations of the vaccine. The antibody titers obtained from the model defined by Eqs (1) and (2) as the area under the curve of *Ab*(*x*, *t*) are compared to the raw data^17^. We found that this variant of the model is able to account for the patterns appearing in the data—as can be seen from Fig. S1, this is also true for model 0, which does not treat NA- and HA-specific antibodies separately. The vaccine formulations with squalene-containing adjuvants result in antibody responses surpassing the detection limit within the first week post immunization, and that remain well above this level for an extended period of time. This is not true for the vaccine containing Diluvac and the non-adjuvanted vaccine, which require the immunization boost to achieve sustained responses above detection level. We also see that the response decays and approaches the detection limit towards 130 days post immunization, which does not happen in the cases of MF59- and AS03-adjuvanted formulations.

Model B, on the other hand, fails to properly capture the decay in functional antibody titers, as can be seen from Fig. S2. At the same time, this has the effect of pulling the immunogenicities towards lower values, in order to compensate for the fast proliferation of B cells at longer times; the resulting dynamics for antibody titers fails to account for the rapid mounting of the response after the first dose, due to the effective decrease in antibody production rates driven by *γ*_NA_ and *γ*_HA_—see Table S2. This suggests that our modulating function needs to play a role on all of the processes involved in the proliferation of the B cell population, rather than only have an effect over those representing recruitment of naive or memory cells.

An additional variant of the model was considered, corresponding to model A for the non-adjuvanted formulation of the vaccine, but letting the adjuvants affect the antigen immunogenicities only; that is, we chose *β*_*Ab*_ = 1, effectively removing a free parameter from the model. This simpler version of adjuvant influence, which we denote by model C, did not reproduce the behavior observed in the data—see Table S3 and Fig. S3. In order to compensate for the absence of boost in the protein-independent rate of antibody production, larger immunogenicity boosts are required. The subsequent shift in weighted affinity results in a much larger selection effect in B cell proliferation, ultimately yielding an excessive rate of Ab production immediately after each of the immunizations.

### B cell and antibody distributions in shape space

**Figure 4:**
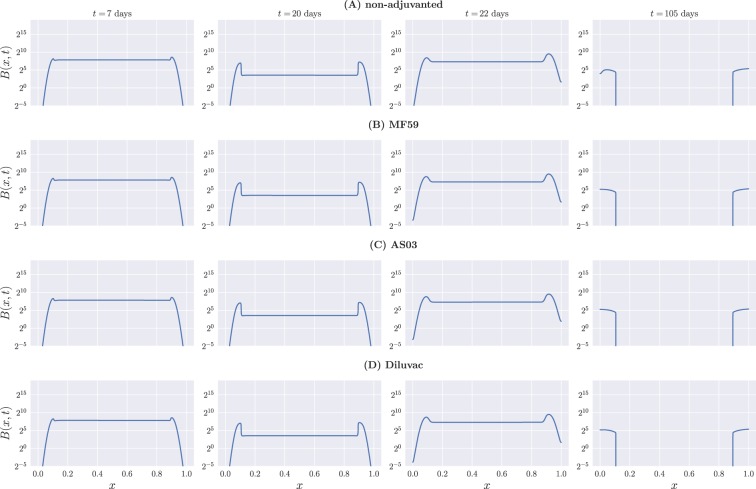
B cell profiles at different times for model A. (**A**) Non-adjuvanted case; (**B**) MF59; (**C**) AS03; (**D**) Diluvac. Each column represents a snapshot of the B cell distribution in shape space at the time stated above the top panel. By construction, differences between the adjuvanted and non-adjuvanted formulations are not clearly discernible here, due to the fact that the immunogenicity-weighted affinity has little effect on B cell dynamics, and the unweighted affinity takes a more important role.

**Figure 5:**
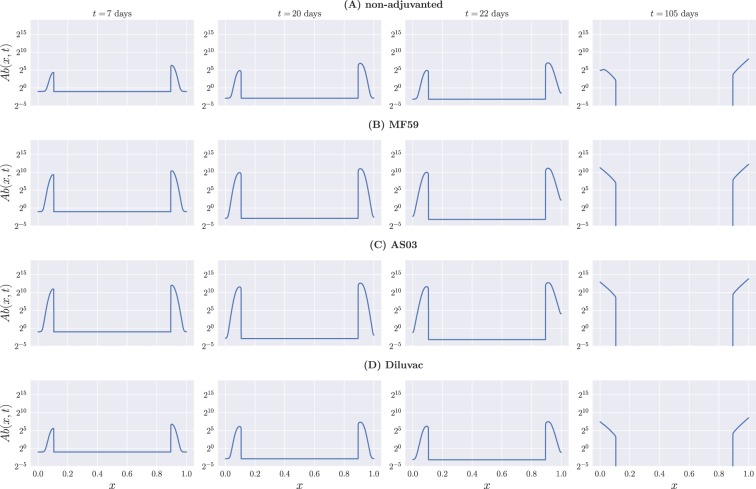
Antibody profiles at different times for model A. (**A**) Non-adjuvanted case; (**B**) MF59; (**C**) AS03; (**D**) Diluvac. Each column represents a snapshot of the Ab distribution in shape space at the time stated above the top panel. In contrast to Fig. 4, here the effects of the adjuvants are clearly visible: we observe the shifts in the scale of the Ab response, much larger in the case of squalene-containing adjuvants, as well as a leveling of the relative heights of the left- and right-most portions of Ab distribution with respect to the non-adjuvanted case, corresponding to the NA- and HA-specific responses, respectively.

Focusing on model A, we next looked directly at the profiles of *B*(*x*) and *Ab*(*x*) at specific times for the four different vaccine formulations. This is shown in Figs 4 and 5 for B cells and antibodies, respectively. We show snapshots of the B cell and Ab distributions at the end of the first week, the end of the third week before boost, the beginning of the fourth week, and the end of the fifteenth week.

As expected from the model construction, the profile of B cells is initially characterized by the influx of naive cells represented by *H*(*x*). As time progresses, mutations widen the profile, and high-affinity cells proliferate faster and decay more slowly than low-affinity ones, resulting in the distribution caving towards the center of the interval. When the GC is no longer active and the naive cell influx has stopped, we are left solely with high-affinity, slowly-decaying B cells.

**Figure 6:**
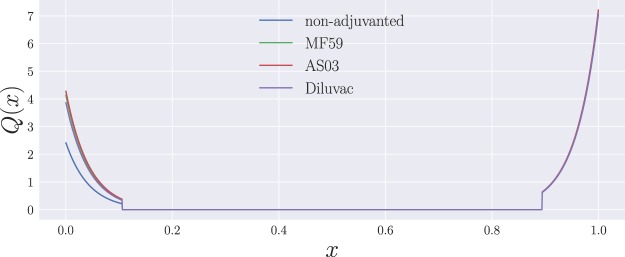
The weighted binding affinity, *Q*(*x*), as a function of vaccine formulation for model A.

Since the dynamics of B cells depends, for the most part, on their unweighted binding affinity—with weighted affinity having an evident influence only towards the edges of the interval, due to the recruiting of ‘memory’ cells—we do not clearly observe the effects of adjuvants on their overall distribution. This can, however, be seen from the antibody profiles in Fig. 5, where we find that the relative heights of the left- and right-hand side peaks of the distribution on the one-dimensional shape space for the adjuvanted vaccine formulations tend to be more even that for the non-adjuvanted vaccine throughout the whole process. This is a direct consequence of the adjuvant-driven boost in glycoprotein immunogenicities, which is primarily directed towards *γ*_NA_, while *γ*_HA_ remains essentially unchanged, as can be seen from Table 2—see also Fig. 6, showing the resulting weighted binding affinity *Q*(*x*) for all vaccine formulations.

The most striking difference between base and adjuvant-boosted parameters corresponds to the rate of antibody production $\tilde{k}$—represented by *β*_*Ab*_—with values tens of times larger for the squalene-containing formulations—over 50 in the case of AS03—as shown in Table 2. This is also the parameter that clearly discriminates between squalene-containing adjuvants and Diluvac, with the latter only resulting in a lower than two-fold increase in antibody production rate.

Finally, from Fig. 5 we note that the effect of the boost immunization on antibody titers is strongest towards the very high-affinity edges of the interval, *x* = 0 and *x* = 1, and not so easily observable towards the peaks of the distribution. This is consistent with the higher rate of proliferation of fully mature ‘memory’ B cells, which also benefit more from the boost that their low-affinity counterparts, as seen in Fig. 4.

### Protein-specific antibodies

Furthermore, we can calculate the total titer of antibodies with non-zero binding affinity towards NA (resp. HA), but zero binding affinity towards HA (resp. NA) at a given moment in time as

15 $$A{b}_{{\rm{NA}}}(t)={\int }_{0}^{{d}_{{\rm{\max }}}}\,{\rm{d}}x\,Ab(x,t),$$

16 $$A{b}_{{\rm{HA}}}(t)={\int }_{1-{d}_{{\rm{\max }}}}^{1}\,{\rm{d}}x\,Ab(x,t).$$

**Figure 7:**
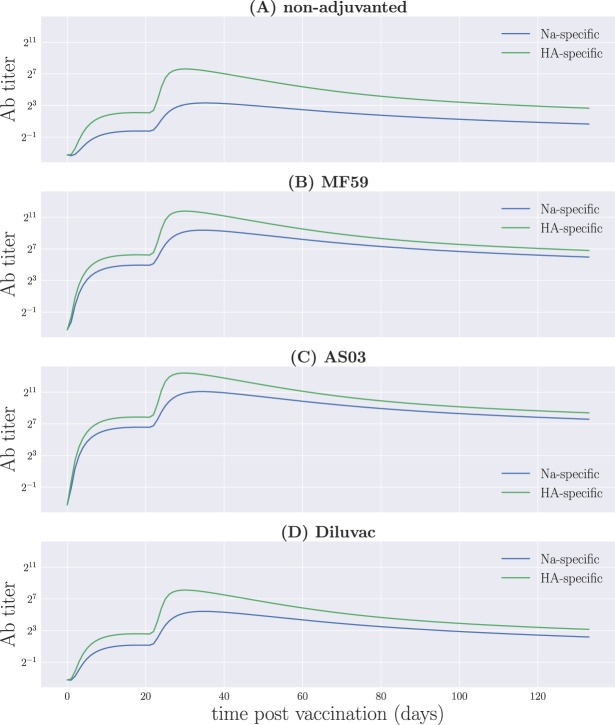
Protein-specific antibody titers for the different vaccine formulations for model A. (**A**) Non-adjuvanted case; (**B**) MF59; (**C**) AS03; (**D**) Diluvac.

This allows us to clearly visualize the kinetics of the NA- or HA-specific antibodies in the system, as illustrated in Fig. 7.

**Figure 8:**
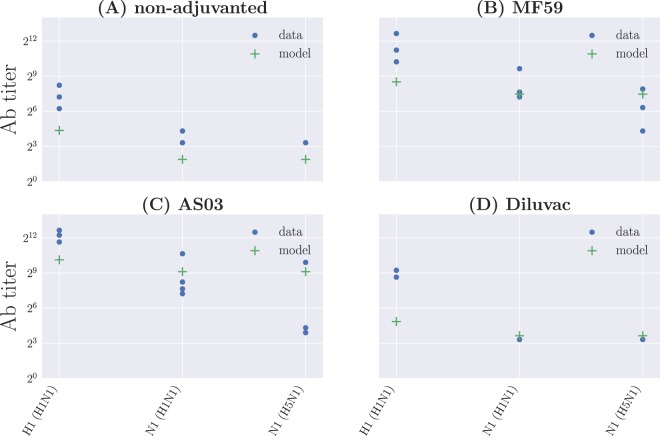
Qualitative comparison between total protein-specific antibody titers from the data and the results for the functional antibody titers from model A. Broadly speaking, the patterns in the data are reproduced, albeit with consistent biases in the predictions due to the fact that we are looking at total Ab titers, and we lack a means to determine a functional Ab fraction from this total. In addition, the model as it stands does not take into account within-subtype variation, and hence predictions for both N1 are the same in all four cases. (**A**) Non-adjuvanted case; (**B**) MF59; (**C**) AS03; (**D**) Diluvac.

These dynamics may be compared, albeit only qualitatively, to the data for total antibody titers against different HA and NA proteins obtained from samples collected at day 76 post immunization^17^. The proteins correspond to H1 (H1N1), H3 (H3N2), H5 (H5N1), H7 (H7N9), H9 (H9N2), N1 (H1N1), N1 (H5N1), and N2 (H3N2); in this case, we consider explicitly only the H1 and both N1 proteins, since these are the ones contained in the vaccine, and assume all other antibody titers to be below the detection level. The results for *Ab*_NA_(*t* = 76) and *Ab*_HA_(*t* = 76) are compared to the data in Fig. 8, and we see that in very broad terms the trends in the data are somewhat reproduced. However, as one may expect, the model in its current version requires a means to determine the proportion of functional antibodies in total antibody titers from the original output, in order to permit a quantitative comparison.

## Discussion

We have presented a model of antibody production dynamics in response to vaccination with adjuvanted and non-adjuvanted influenza vaccines. Our focus was to construct a parsimonious description of antibody dynamics that is capable of reproducing experimentally observed behavior. With this in mind, we derived a deterministic one-dimensional representation of the distribution of B cell and antibody lineages in shape space. Two different variants of the base, non-adjuvanted model were explored: model A, considering a modulation in B cell growth as a whole; and model B, consisting in a modulation of cell recruitment into the GC only. The results obtained favor a picture consistent with a broader regulation inside the GC itself, as the decay in B cell numbers is not fast enough to compensate for their proliferation and cannot, by itself, account for the decay in antibody titers observed at later time points^17^.

For the adjuvanted formulations of the vaccine, the specific adjuvants studied were MF59, AS03 and Diluvac Forte. The latter is a veterinary approved adjuvant containing vitamin E in watery suspension^37^. MF59 and AS03, on the other hand, are oil-in-water emulsions based on squalene oil, which are approved for use in humans and count with extensive evidence for their properties as adjuvants. Squalene emulsions stimulate immune cell migration to the injection site and antigen uptake, resulting in adjuvanted vaccines that yield increased antibody titers with respect to their non-adjuvanted counterparts—see, *e*.*g*., refs^38–40^. At the same time, they have shown effects on cross-reactivity^41,42^ and reduction of virus replication and transmission^43^, and are well tolerated, with a strong safety record^38,44^. These features make squalene-based adjuvants excellent candidates for antigen-sparing vaccination strategies.

We have introduced the influence of adjuvants in the model as a boost in glycoprotein immunogenicities, and found that all adjuvanted formulations of the vaccine result in enhanced antibody response against NA. We also found that the boost in immunogenicities alone—illustrated by model C, shown in Table S3 and Fig. S3—is not sufficient to account for the observed titers, and an additional parameter representing an adjuvant-driven boost in the overall rate of antibody production in the GC is required to yield quantitative agreement with the data. The latter influences both the NA- and HA-specific responses in the same way and is, in fact, the strongest effect of the adjuvants on the resulting antibody titers. In other words, there exist a qualitative change in the adjuvant-driven antibody response, brought about by the skewed boost in the reaction towards the NA protein. However, the most important effect induced by the adjuvants, and the key feature that separates Diluvac from squalene-containing adjuvants, is the sheer scale of the resulting overall antibody response, irrespective of protein specificity. It is important to note that model A, with *β*_*Ab*_ being a free parameter, is the minimal model that reproduces the observed experimental behavior for the adjuvanted vaccine formulations in relation to model C given the assumptions and fixed parameters governing the non-adjuvanted case. In other words, we do not rule out the possibility that a different formulation for the base, non-adjuvanted model might work in conjunction with the assumptions underpinning model C, *i*.*e*., that the adjuvants have an effect on protein immunogenicities only.

**Figure 9:**
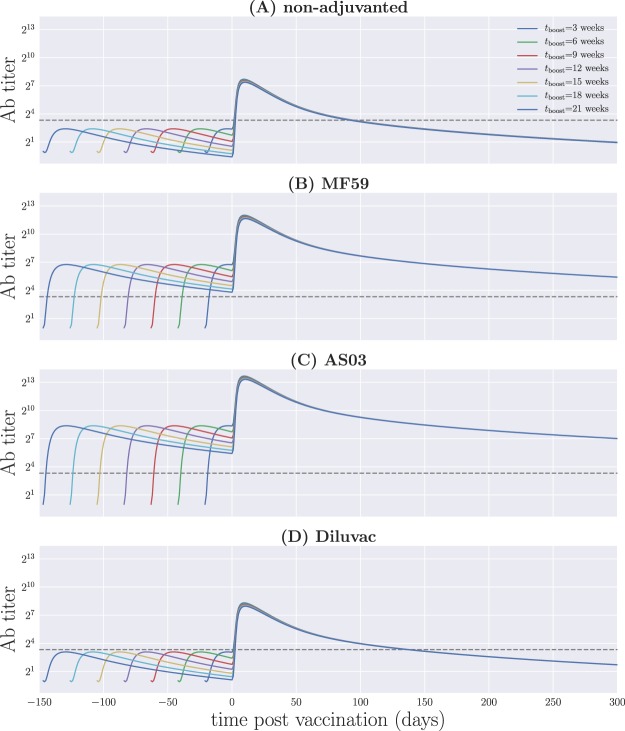
Antibody titer from model A for different timings of the second dose. (**A**) Non-adjuvanted case; (**B**) MF59; (**C**) AS03; (**D**) Diluvac. In all cases, the gray, dashed line shows the detection limit for neutralizing antibodies.

The resulting dynamics from the model can be explored under different conditions from those in Schmidt *et al*.^17^. It has been observed in ferrets that, while a second dose of the vaccine is required in order to obtain high and sustained immune responses, its timing does not have a significant impact on their overall behavior for the case of AS03^45^. Similar results have been observed in clinical trials of MF59-adjuvanted influenza vaccines, with between-dose intervals of up to six weeks for adults^46^, and high antibody titers up to one year following vaccination with four weeks between doses in young children^47^. Using the best fit parameters from Table 2, we can simulate Eqs (1) and (2) with different values of *t*_boost_ and assess the model output for all vaccine formulations, as shown in Fig. 9.

We observe that, in the context of model A, the long-term behavior of functional antibody titers is largely unaffected by the timing of the immunization boost for all four vaccine formulations; only the maximum titer achieved shows a small variation, being larger when the initial vaccination and the boost are more closely spaced. Furthermore, a strong response is not maintained for the Diluvac- and non-adjuvanted vaccines. In contrast, the squalene-based adjuvants result in antibody titers well above the detection limit for extended periods after the boost. This is consistent with previous findings^45–47^, and suggests that vaccines formulated with squalene-containing adjuvants are generally robust to changes in the interval between the first and second doses. Further experiments involving AS03 and MF59 in different prime-boost schedules would help elucidate this claim.

An interesting outcome resulting from the fact that model A is based on distributions in shape space—in contrast to model 0, which treats B cells and Abs as scalar variables—corresponds to the option of tracking the dynamics of the NA- and HA-specific immune responses independently of one another, as we have shown in Fig. 7. This opens up the possibility of exploring the introduction of additional virus strains, and determining the antibody response against different glycoprotein variants. In particular, one may assess the effects of the qualitative shift on protein-specific responses induced by the adjuvanted formulations on the antibody response towards strains carrying the same NA subtype, and their implications for cross-reactive immunity. However, within the one-dimensional representation we have adopted for shape space, it is not immediately clear how to include different variants of NA and HA. This is most evident from Fig. 8, where we have not made distinctions between different NA proteins belonging to the same subtype.

A possible way of extending the model to accommodate different HA and NA proteins may be to adopt a picture of an antibody landscape such as that from Fonville *et al*.^30^, in which different virus strains are mapped to a one-dimensional space, but doing so protein-wise. In other words, employing a two-dimensional space and mapping HA and NA independently to positions along either dimension, such that HA- and NA-specific immune responses give rise to separate landscapes. The model may be further extended by coupling it to additional equations describing the time evolution of within-host influenza dynamics, such as the target-cell limited model described in Baccam *et al*.^48^. This would allow us to assess the effects of the different vaccine formulations on post-challenge viral load dynamics; additional experiments could then be conducted in order to test these results.

As a final note, we must remark on an important limitation of our work. While we have attempted to derive a model which is as minimal as possible, we are nevertheless interested in being able to account for Ab distribution along the shape-space. As a consequence, we are left with a large number of parameters given the size of the dataset and, in order to focus on the relative Ab responses between adjuvanted and non-adjuvanted vaccine formulations, we have chosen to rely on assumptions and fix parameter values whenever possible. In particular, we have fixed most of the parameters that are part of the base, non-adjuvanted model, and that we do not expect to change for the adjuvanted formulations. We have taken these from previous estimations and assumptions^27,32–34^, while others have been fixed either because we expect them to have a negligible effect—such as setting *d*_min_ = 0—or rescale one another in the non-adjuvanted case; the latter is the case of the height of the naive pool, $\tilde{H}$, and the variance *ν*, which determines how fast the low-affinity B cells diffuse into the regions of higher affinity. Nevertheless, we expect that choosing a working combination of values for $\tilde{H}$ and *ν* does not represent a problem for the purpose of comparing different vaccine formulations, since these are kept constant throughout the comparison. Another obvious rescaling pair corresponds to the protein immunogenicities, which compete directly against one another in antibody production in order to match the model output to the experimental data. Here we have not fixed either of them, but have rather used the fact that HA is known to be more immunogenic than NA^13^ and that the neutralizing response is skewed towards HA^7^ in order to constrain the possible values of *γ*_NA_ while giving more freedom of movement to *γ*_HA_. This biologically inspired choice constitutes an assumption of the model, and it is not a direct result of the parameter estimation.

A sensitivity analysis reveals that, for the base model, changes to the rate of decay in GC activity, *μ*, and to the size of the affinity region, *d*_max_, have the largest quantitative effect on the outcome of the model—see Fig. S4(C,D). At the same time, the immunogenicity of NA is the parameter with the least impact—Fig. S4(E). Changes to the parameters controlling the effects of the adjuvants—*β*_NA_, *β*_HA_, and *β*_*Ab*_—result in similar outcomes, with the Ab titer being to some extent more robust to changes in *β*_NA_, as shown in Fig. S5. Additionally, we see that, as anticipated, the fixed parameters $\tilde{H}$ and *ν* have a similar *qualitative* effect on the outcome of the model, so that larger values of one of them will very require smaller values of the other; this is illustrated in Fig. S6, showing the sensitivity function—as defined in Soetaert & Petzoldt^49^—of the two parameters for the log_2_ of the Ab titer in the non-adjuvanted case. We note, however, that *ν* has a larger impact on the resulting titers after the boosting time. This can be explained by the fact that $\tilde{H}$ is only relevant in the low-affinity region of the shape space, while *ν* controls the rate of diffusion towards higher affinities in the entire interval, and the boost has a stronger effect on high-affinity B cells.

## Electronic supplementary material

Supplementary Material
