# Supplementary material for: Adjuvanted influenza vaccine dynamics
Source: Sci Rep. 2019 Jan 11;9:73. doi: 10.1038/s41598-018-36426-9 (PMC6329778; doi:10.1038/s41598-018-36426-9)
Supplement: Supplementary file 1 — Supplementary Material [file 41598_2018_36426_MOESM1_ESM.pdf]

Adjuvanted influenza vaccine dynamics

César Parra-Rojas[1], Veronika von Messling[2], and Esteban A. Hernandez-Vargas[1,*]

[1]Frankfurt Institute for Advanced Studies, 60438 Frankfurt am Main, Germany
[2]Veterinary Medicine Division, Paul-Ehrlich-Institut, Federal Institute for Vaccines and Biomedicines, 63225 Langen, Germany
[*]vargas@fias.uni-frankfurt.de

Supplementary Models

Model 0

This is the simplified version of model A without protein specificity, where B and Ab are scalar variables.

$$\frac{\mathrm{d}B}{\mathrm{d}t} = G(t)\,[H + QB + \tilde{r}B] - \frac{g_B^{(0)}}{1 + Q g_B^{(1)}} B - \frac{\tilde{r}}{K} B^2 \tag{S1}$$

$$\frac{\mathrm{d}Ab}{\mathrm{d}t} = \alpha B - g_{Ab} Ab \tag{S2}$$

Linking the dynamics above with Eqs. (1)–(2) of the main text, the free parameters in the non-adjuvanted case are the influx of naive cells H, the effective affinity Q, the rate of Ab production α and the rate of GC decay μ—see $G(t)$ in Eq. (7) of the main text. The adjuvanted formulations are associated with boosts in Q and α:

$$Q \to \beta_Q Q, \tag{S3}$$

$$\alpha \to \beta_\alpha \alpha \tag{S4}$$

Model B

This model differs from model A in the way the modulation function G enters the dynamics. In this case, B cells continue to proliferate as long as a non-zero population remains, and only activation/recruitment terms are affected by a decay in GC activity.

$$\begin{aligned}\frac{\partial B(x,t)}{\partial t} &= G(t)\,[\sigma_N H(x) + \sigma_M Q(x) B(x,t)] + \tilde{r} B(x,t) + D \frac{\partial^2 B(x,t)}{\partial x^2} \\ &\quad - g_B(x) B(x,t) - \frac{\tilde{r}}{K}\,[B(x,t)]^2 .\end{aligned} \tag{S5}$$

$$\frac{\partial Ab(x,t)}{\partial t} = \delta\,\tilde{k}\,Q(x) B(x,t) - g_{Ab} Ab(x,t). \tag{S6}$$

Model C

The model itself is the same as model A, Eqs. (1)–(2) from the main text. The difference lies in the choice of free parameters representing the influence of adjuvants on the system. While model A considers that both glycoprotein immunogenicities and the overall rate of antibody production are boosted by the adjuvants, model C only takes the former into account. The boosts are as follows:

$$\gamma_{\mathrm{NA}} \to \beta_{\mathrm{NA}}\,\gamma_{\mathrm{NA}}, \tag{S7}$$

$$\gamma_{\mathrm{HA}} \to \beta_{\mathrm{HA}}\,\gamma_{\mathrm{HA}}. \tag{S8}$$

Compare this to Eqs. (9)–(11) of the main text, for the case of model A.

Supplementary Tables and Figures

Tables

formulation	H (0.1, 1000.0)	Q (0.01, 50.0)	α (1.0, 5.0)	μ (0.1, 2.0)	β_Q (1.0, 100.0)	β_α (1.0, 100.0)
non-adjuvanted	0.2289	0.0286	1.3424	0.5461	–	–
MF59	–	–	–	–	5.4123	6.6174
AS03	–	–	–	–	11.0181	9.6754
Diluvac	–	–	–	–	1.7113	1.0

Table S1. Best fit parameters obtained for model 0, Eqs. (S1)–(S2). The bounds used in the estimation procedure are shown under each parameter.

formulation	$d_{\max}$ (0.1, 0.3)	μ (0.2, 1.0)	γ_{NA} (0.1, 2.5)	γ_{HA} (0.1, 7.5)	β_{NA} (1.0, 5.0)	β_{HA} (1.0, 5.0)	β_{Ab} (1.0, 60.0)
non-adjuvanted	0.2965	0.2183	0.1707	0.1238	–	–	–
MF59	–	–	–	–	2.2628	1.7041	18.1582
AS03	–	–	–	–	4.6510	1.6643	24.9760
Diluvac	–	–	–	–	2.5424	1.1605	1.0026

Table S2. Best fit parameters obtained for model B, Eqs. (S5)–(S6). The bounds used in the estimation procedure are shown under each parameter.

formulation	β_{NA} (1.0, 100.0)	β_{HA} (1.0, 100.0)
MF59	8.2604	2.9283
AS03	11.5455	4.5064
Diluvac	2.4073	1.0002

Table S3. Best fit parameters obtained for model C, Eqs. (1)–(2) from the main text, with Eqs. (9)–(11) replaced by Eqs. (S7)–(S8)—*i.e.*, $\beta_{Ab} \equiv 1$. The bounds used in the estimation procedure are shown under each parameter. Parameters for the non-adjuvanted case correspond to model A.

Figures

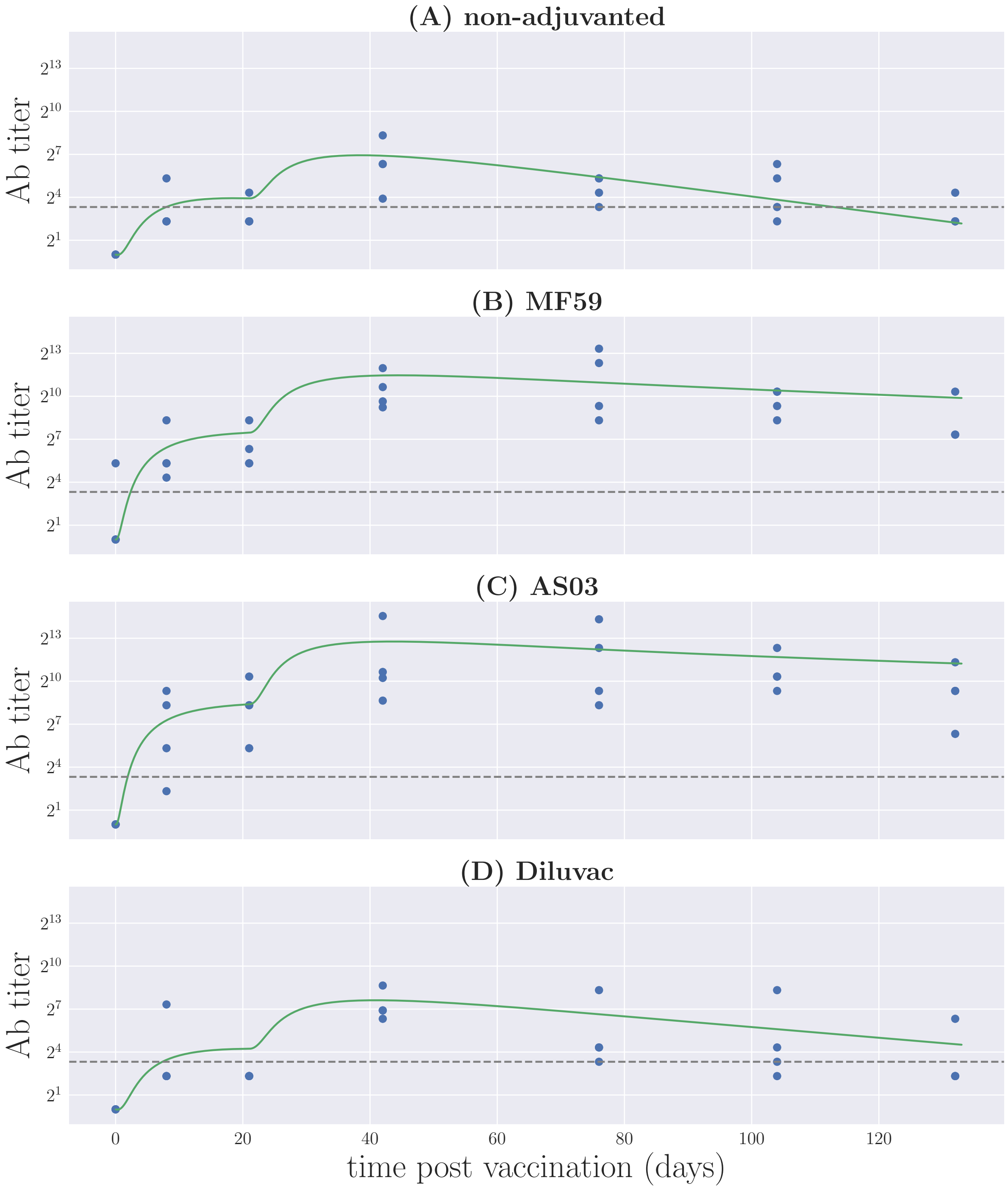

Figure S1. The raw data for virus-neutralizing antibody titers, compared to the result for $Ab(t)$ from model 0 for the parameter values from Table S1. (A) Non-adjuvanted case; (B) MF59; (C) AS03; (D) Diluvac. In all cases, the gray, dashed line shows the detection limit for neutralizing antibodies.

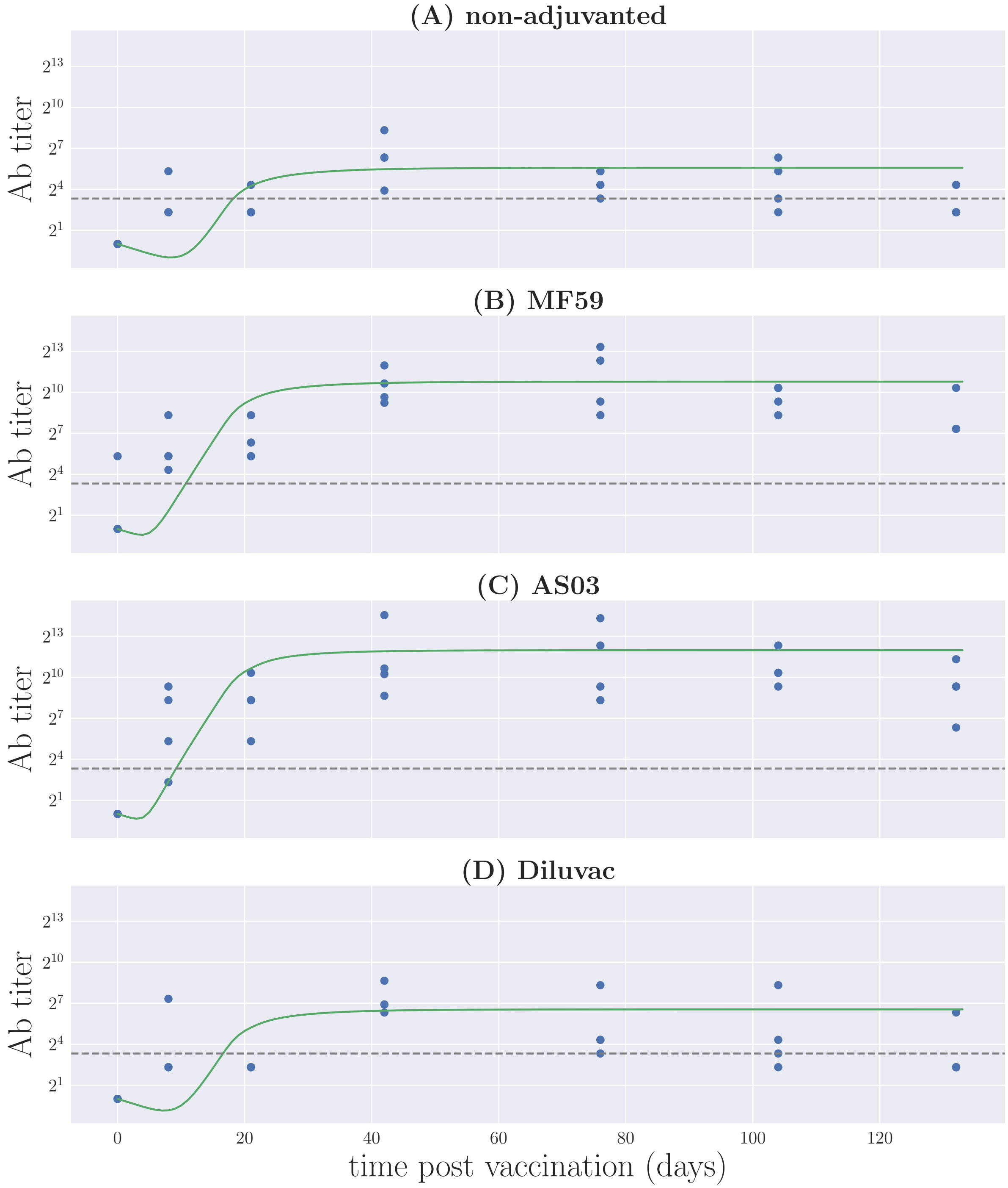

Figure S2. The raw data for virus-neutralizing antibody titers, compared to the area under the curve of $Ab(x,t)$ resulting from model B for the parameter values from Table S2. (A) Non-adjuvanted case; (B) MF59; (C) AS03; (D) Diluvac. In all cases, the gray, dashed line shows the detection limit for neutralizing antibodies.

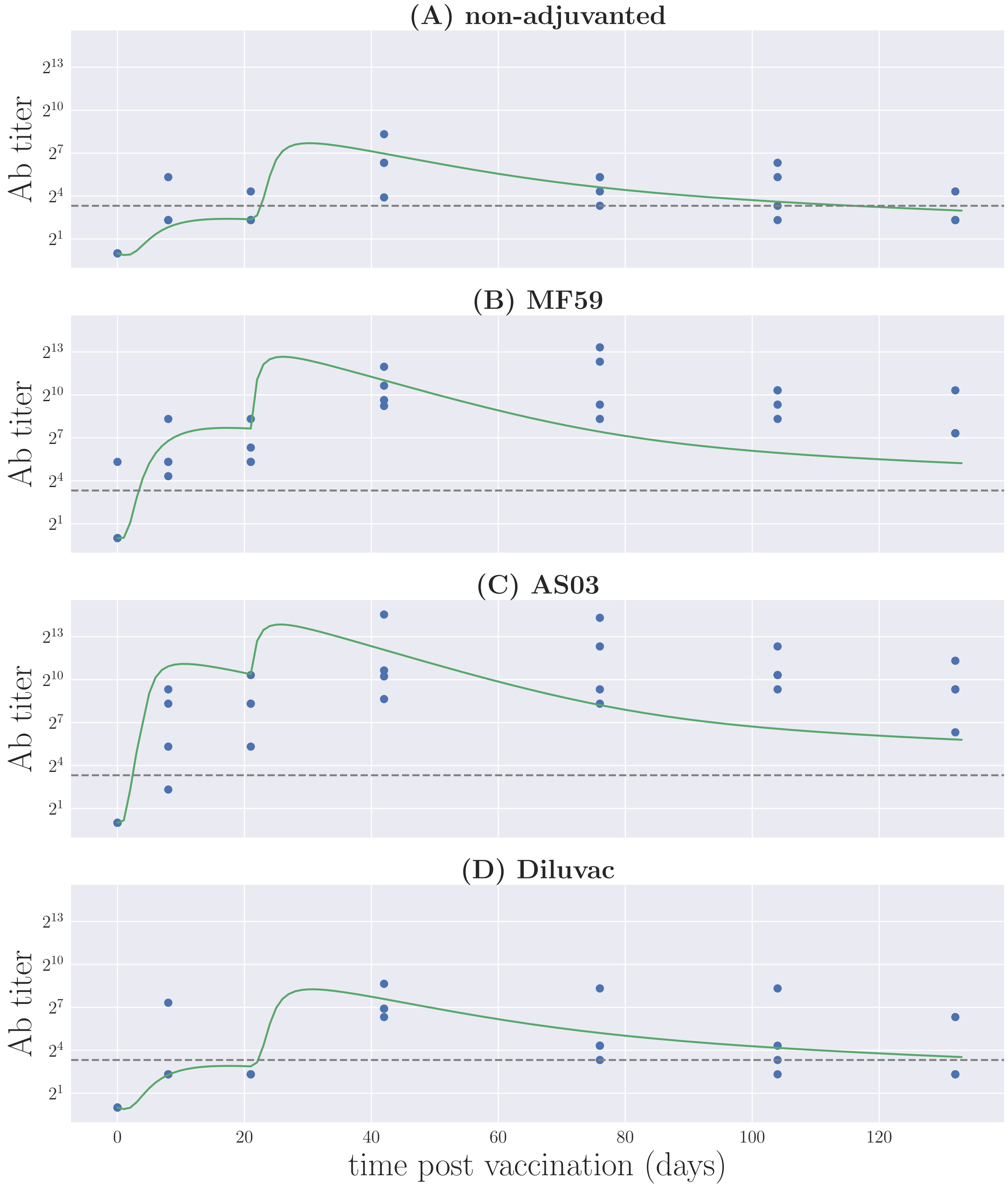

Figure S3. The raw data for virus-neutralizing antibody titers, compared to the area under the curve of $Ab(x,t)$ resulting from model C for the parameter values from Table S3. (A) Non-adjuvanted case; (B) MF59; (C) AS03; (D) Diluvac. In all cases, the gray, dashed line shows the detection limit for neutralizing antibodies.

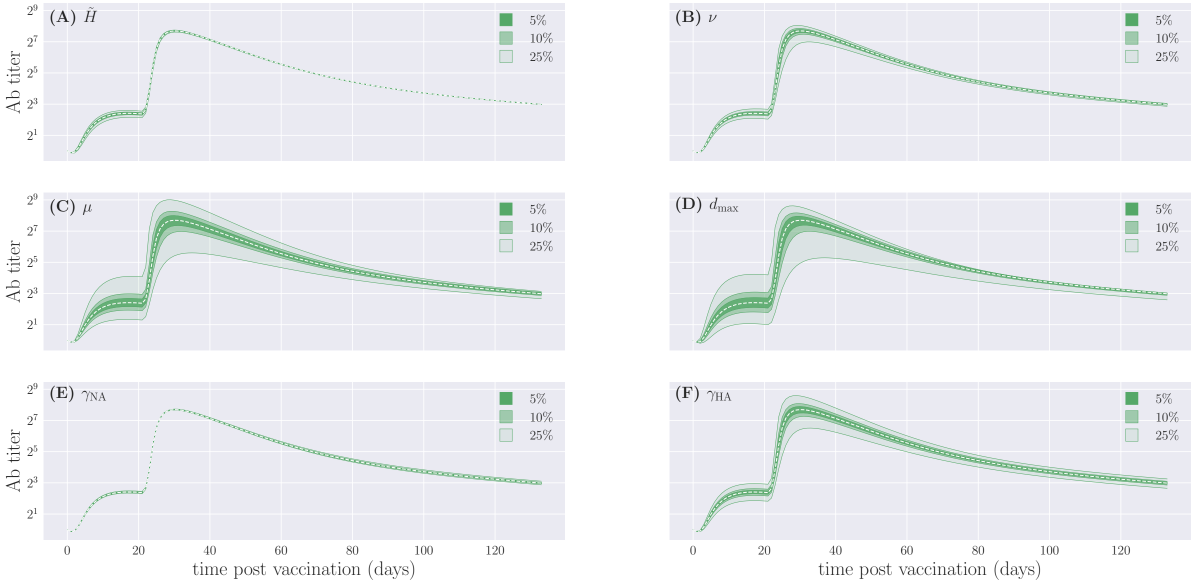

Figure S4. Ab titer for the non-adjuvanted formulation resulting from changes to the base parameters. The white, dashed line corresponds to the titer at the nominal value from Tables 1 ($\tilde{H}$ and ν) and 2 (all the rest) of the main text.

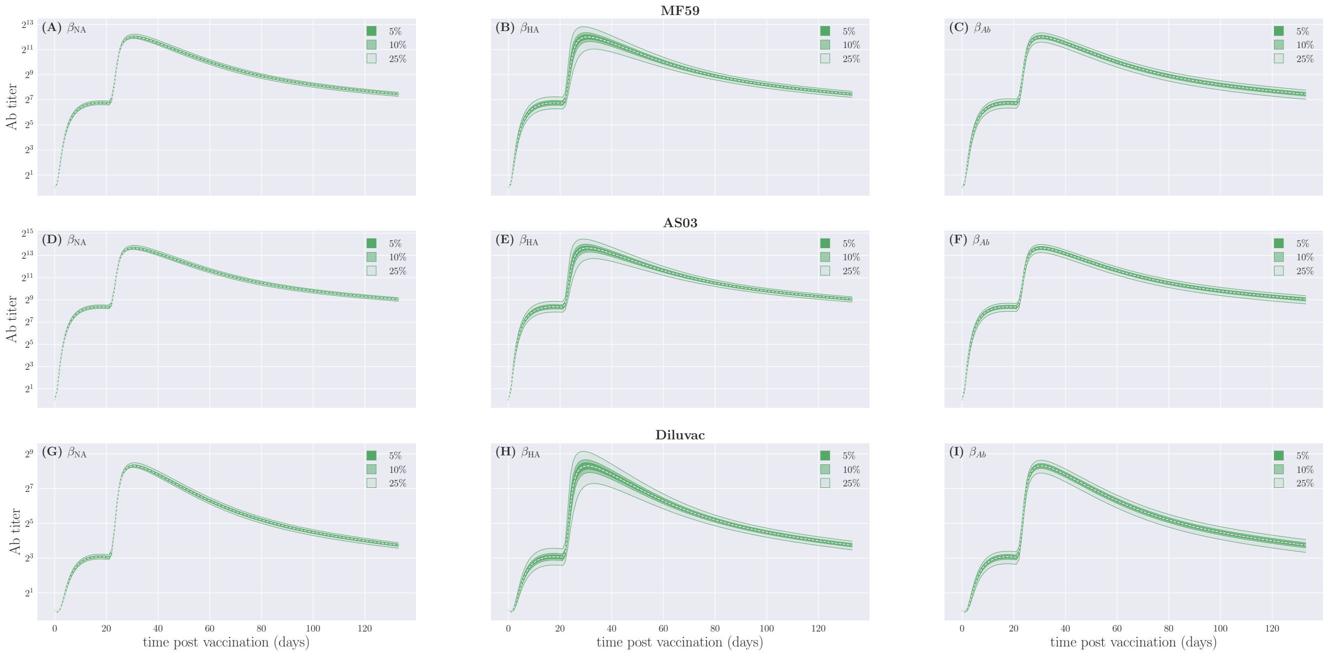

Figure S5. Ab titer for the adjuvanted formulations resulting from changes to the boosting parameters. The white, dashed line corresponds to the titer at the nominal value from Table 2 of the main text.

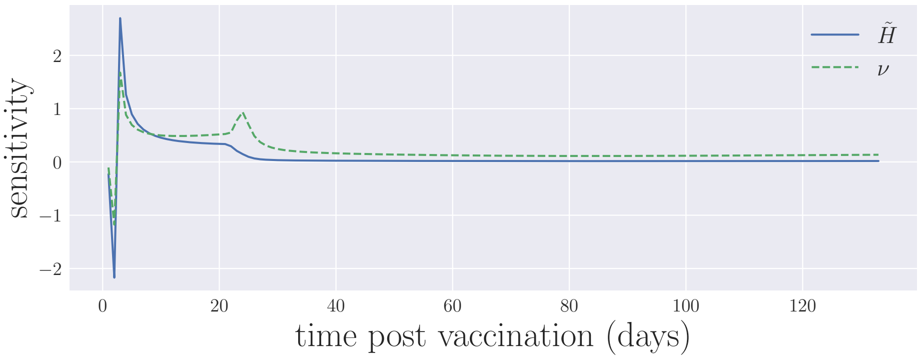

Figure S6. Sensitivity functions of $\tilde{H}$ and ν for the $\log_2$ of the resulting Ab titer in the non-adjuvanted formulation.
